# BGLM: big data-guided LOINC mapping with multi-language support

**DOI:** 10.1093/jamiaopen/ooac099

**Published:** 2022-11-25

**Authors:** Ke Liu, Martin Witteveen-Lane, Benjamin S Glicksberg, Omkar Kulkarni, Rama Shankar, Evgeny Chekalin, Shreya Paithankar, Jeanne Yang, Dave Chesla, Bin Chen

**Affiliations:** Department of Biostatistics, School of Public Health, Cheeloo College of Medicine, Shandong University, Jinan, Shandong, China; Department of Pediatrics and Human Development, College of Human Medicine, Michigan State University, Grand Rapids, Michigan, USA; Office of Research, Spectrum Health, Grand Rapids, Michigan, USA; Icahn School of Medicine at Mount Sinai, The Hasso Plattner Institute for Digital Health at Mount Sinai, New York City, New York, USA; Department of Genetics and Genomic Sciences, Icahn School of Medicine at Mount Sinai, New York City, New York, USA; Office of Research, Spectrum Health, Grand Rapids, Michigan, USA; Department of Pediatrics and Human Development, College of Human Medicine, Michigan State University, Grand Rapids, Michigan, USA; Department of Pediatrics and Human Development, College of Human Medicine, Michigan State University, Grand Rapids, Michigan, USA; Department of Pediatrics and Human Development, College of Human Medicine, Michigan State University, Grand Rapids, Michigan, USA; College of Literature, Science, and the Arts, University of Michigan, Ann Arbor, Michigan, USA; Office of Research, Spectrum Health, Grand Rapids, Michigan, USA; Department of Obstetrics, Gynecology and Reproductive Biology, College of Human Medicine, Michigan State University, Grand Rapids, Michigan, USA; Department of Pediatrics and Human Development, College of Human Medicine, Michigan State University, Grand Rapids, Michigan, USA; Department of Pharmacology and Toxicology, College of Human Medicine, Michigan State University, Grand Rapids, Michigan, USA

**Keywords:** big data, LOINC code mapping, electronic health records, multi-language support

## Abstract

**Motivation:**

Mapping internal, locally used lab test codes to standardized logical observation identifiers names and codes (LOINC) terminology has become an essential step in harmonizing electronic health record (EHR) data across different institutions. However, most existing LOINC code mappers are based on text-mining technology and do not provide robust multi-language support.

**Materials and methods:**

We introduce a simple, yet effective tool called big data-guided LOINC code mapper (BGLM), which leverages the large amount of patient data stored in EHR systems to perform LOINC coding mapping. Distinguishing from existing methods, BGLM conducts mapping based on distributional similarity.

**Results:**

We validated the performance of BGLM with real-world datasets and showed that high mapping precision could be achieved under proper false discovery rate control. In addition, we showed that the mapping results of BGLM could be used to boost the performance of Regenstrief LOINC Mapping Assistant (RELMA), one of the most widely used LOINC code mappers.

**Conclusions:**

BGLM paves a new way for LOINC code mapping and therefore could be applied to EHR systems without the restriction of languages. BGLM is freely available at https://github.com/Bin-Chen-Lab/BGLM.

## INTRODUCTION

Laboratory test data stored in electronic health record (EHR) systems are valuable resources for clinical studies. Although more and more EHR datasets are being utilized for research, each institution uses its local coding system to refer to lab tests, and such inconsistency in lab test reference creates a significant barrier for downstream integrative analysis. Logical observation identifiers names and codes (LOINC) provides a universal coding standard for lab tests and is widely used in EHR data standardization processes.^1^ To enable lab test data to be integrable with data from other institutions, mapping internally used lab test local codes to LOINC codes is necessary.

Since the number of lab test local codes stored in an EHR system is large, manual mapping is time-consuming and not feasible at scale. Multiple tools have been developed to automate and accelerate the LOINC code mapping process.^2–4^ Although different in detail, most of them conduct the mapping based on the similarity between text-formatted metadata (such as lab test names, descriptions) of local codes and LOINC codes. These automatic LOINC code mappers have achieved high success; however, a few challenges remain and need to be addressed.

First, existing methods have limited multi-language support. Currently, almost all LOINC code mappers only accept English texts as their input. It is time-consuming for non-native English speakers to accurately translate the texts stored in their parent language to English and it is even infeasible when performing mapping in large-scale EHR systems.

Second, existing methods do not fully utilize patient data. The EHR system stores not only the metadata of lab tests but also their observed values in patients. The lab test metadata is relatively static and rarely updated while the amount of patient data rapidly increases as long as the EHR system is in use. These accumulated patient data are also valuable resources for LOINC code mapping; however, no strategy has been proposed to leverage them.

Here, we propose a novel big data-guided LOINC code mapper (BGLM), which simultaneously addresses the above two technical challenges. Different from existing methods, BGLM maps local codes to LOINC codes based on distributional similarity. We validated the performance of BGLM with in-house EHR datasets and showed that high mapping precision could be achieved under proper false discovery rate (FDR) control.

## MATERIALS AND METHODS

**Figure 1.**
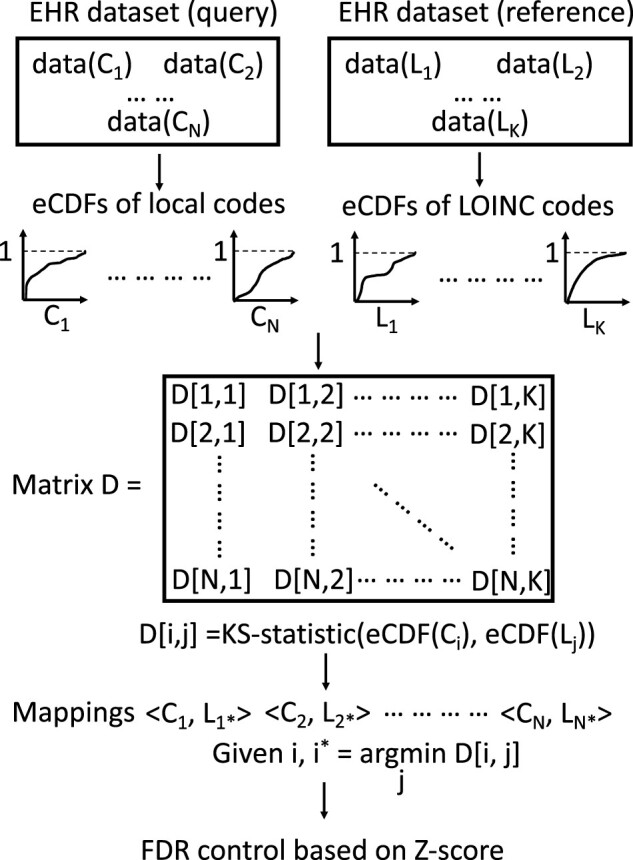
Detailed workflow of BGLM. *C_i_*, the *i*-th local code; *L_j_*, the *j*-th LOINC code; eCDF: empirical cumulative distribution function; and KS-statistic, Kolmogorov–Smirnov test statistic.

The BGLM workflow is composed of three major steps (Figure 1).

### Step I. Construction of empirical cumulative distribution functions

Given an EHR dataset (which we call the “query”), various lab test local codes (denoted as *C*_1_, *C*_2_, … *C_N_*) are needed to be mapped. For each local code, 1000 reading values of the corresponding lab test are randomly drawn from the EHR system to construct its empirical cumulative distribution function (eCDF). Similarly, we construct the eCDFs of LOINC codes (denoted as *L*_1_, *L*_2_, … *L_K_*) using the reference EHR dataset (in which LOINC codes, instead of local codes, are used to refer to lab tests).

### Step II. Generation of mappings

We form a *N* × *K* distance matrix *D* with its entry *D* [*i*, *j*] representing the Kolmogorov–Smirnov test statistic between the eCDFs of *C_i_* and *L_j_*. Then, the LOINC code mapping of *C_i_* is L_i_* where

$$i^{*}=\mathrm{argmin}:\;D[i,j]\;j\in [1,2,\;\ldots \ldots ,\;K].$$

### Step III. Control of FDR

Given *C_i_*, we derive a *Z*-score vector (denoted as *Z*) by scaling the *i*-th row of the distance matrix *D*. If *Z* [i*] (*Z*-score of the mapping <*C_i_*, L_i_*>) is smaller than the predefined cut-off, the mapping <*C_i_*, L_i_*> is accepted; otherwise, it is considered a wrong mapping and dropped.

## RESULTS

The publicly available EHR dataset MIMIC-III was used as the reference to construct eCDFs of LOINC codes.^5^ Our in-house EHR dataset derived from Spectrum Health West Michigan was used as the query. There were 113 local codes with more than 1000 data points and their manually mapped LOINC codes (which are considered as true mappings) were within MIMIC-III. We utilized BGLM to generate the mappings for them and observed that the derived *Z*-scores of correct mappings were significantly lower than those of incorrect mappings (*P*-value = 1.192*e*−08, Supplementary Figure S1a), which provides strong evidence to use the *Z*-score to control FDR. Since there is randomness in our method, we ran BGLM (on the same datasets) 30 times to get an average performance curve (Supplementary Figure S1b). The precision value is 0.33 if all the mappings are accepted; however, it could be boosted to 0.79 using *Z*-score cut-off −5.

**Figure 2.**
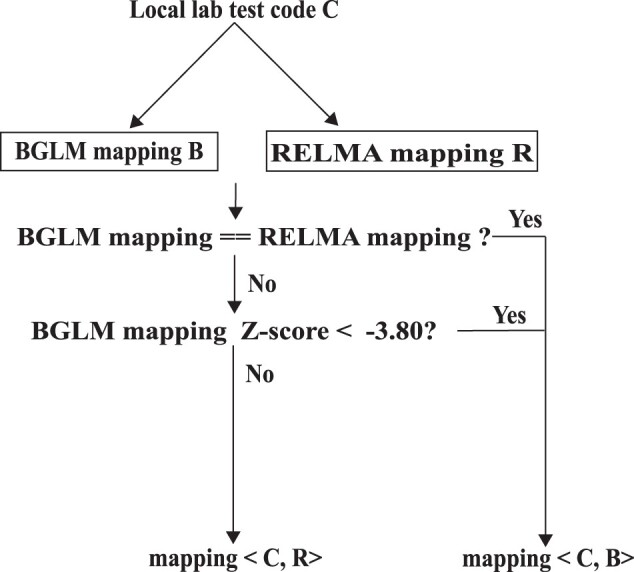
Design of the ensemble LOINC code mapper. Given a local lab test code C, suppose the LOINC mapping generated by BGLM and RELMA are B and R, respectively. If B and R are identical (or the *Z*-score of B is smaller than −3.80), the mapper mapped C to B; otherwise, it mapped C to R.

We also performed the mapping with Regenstrief LOINC Mapping Assistant (RELMA), one of the most widely used LOINC code mappers.^6^ Although the performance of RELMA is better than BGLM (precision = 0.43, Supplementary Figure S1b), it does not provide any criteria to help users control FDR. We therefore designed an ensemble LOINC code mapper based on the rule shown in Figure 2 and found its precision achieved 0.5, suggesting the results of BGLM could be used to improve the mapping quality of RELMA. More details of the ensemble LOINC code mapper are included in the Supplementary Material.

## DISCUSSION AND CONCLUSIONS

The LOINC code mapping in BGLM is solely dependent on distributional similarity and such characteristic enables it to be applied to any EHR systems without considering the internally used language. BGLM paves a new way of LOINC code mapping and achieves ideal performance on our in-house and the public MIMIC-III datasets. Meanwhile, we notice that there are limitations which should be overcome to further enhance BGLM.

First, only the MIMIC-III dataset was used to construct the eCDFs of LOINC codes and this may bring biases to the mapping process. In the future, we will integrate multiple publicly available EHR datasets (such as NIH’s All of Us researcher workbench, UK Biobank, and N3C) to construct more accurate reference eCDFs of LOINC codes, which should improve the performance of BGLM.^7^^,^^8^ Prior to our study, Bradwell et al^9^ proposed a revolutionary data-driven approach to harmonize units and values of quantitative data elements in a large nationally pooled EHR dataset, suggesting the validity and feasibility of such strategy in heterogeneous EHR data harmonization.

Second, the current version of BGLM does not check the consistency of “unit of measure” between local code and its mapped LOINC code, which may result in false positive mappings. We will introduce additional matching processes to overcome such limitation in the next release of BGLM.

## Supplementary Material

ooac099_Supplementary_Data

## Data Availability

The source code and tutorial of BGLM are freely available at https://github.com/Bin-Chen-Lab/BGLM. Our testing in-house data are available upon reasonable request from Spectrum Health.
