# Supplementary material for: BGLM: big data-guided LOINC mapping with multi-language support
Source: JAMIA Open. 2022 Nov 25;5(4):ooac099. doi: 10.1093/jamiaopen/ooac099 (PMC9696745; doi:10.1093/jamiaopen/ooac099)
Supplement: ooac099_Supplementary_Data [file ooac099_supplementary_data.docx]

**Supplementary material**

**Data sources**

The MIMIC-III dataset was downloaded from <https://physionet.org/content/mimiciii>. In total, there are 8301 unique local codes stored in the Epic system of Spectrum Health and we used our in-house SQL scripts to pull out the lab-test data. Of the 8301 local codes, 113 passed our filtering criteria (mapped LOINC code is within MIMIC-III and has more than 1,000 lab-test reading values) and were used to validate BGLM.

According to Glivenko-Cantelli theorem, the empirical cumulative distribution function (eCDF) converges almost surly to true cumulative distribution function as the sample size $n\to\infty$ and that is why we require the number of data points used for mapping can’t be too small (in this manuscript, at least 1,000). Otherwise, BGLM may return mis-leading results due to randomness in the data.

**Mapping local codes to LOINC codes with RELMA**

The RELMA software was download from https://loinc.org/relma/. To map local lab-test codes to LOINC codes, the “Lab Auto Mapper” of RELMA was used. This option uses the test names’ words and suggests probable LOINC matches. The imported file contained lab codes and lab names. The “Maximum number of close LOINC term matches to return” option was set to “1”. “Prefer Common Lab Results” option was checked (Figure S2).

**Design of the ensemble LOINC code mapper**

We tried different z-score cutoff values and found the ensemble LOINC code mapper achieved its highest performance at three values: -3.80, -3.75, and -3.70 (Figure S3).

**Supplementary Figure 1: validation of BGLM.**

(a) Boxplot of Z-scores of LOINC code mappings. Each dot represents a mapping, and the p-value is computed with two-sided Wilcoxon rank-sum test.

(b) The average performance curve of BGLM. The color of a dot represents the cut-off applied on Z-score, the x-axis represents the fraction of mappings with Z-score lower than the cut-off, and y-axis represents the precision. The red dot represents the performance of RELMA.


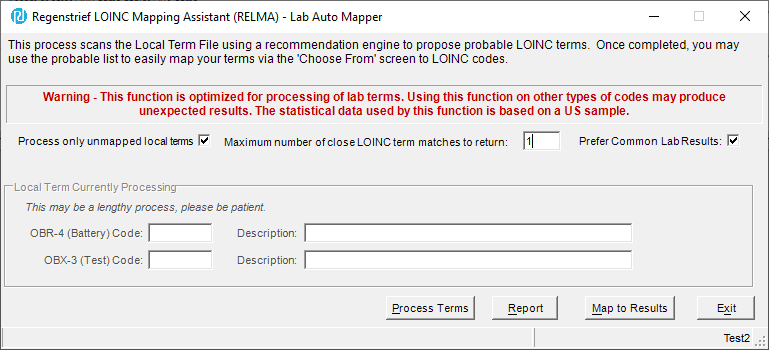

**Supplementary Figure 3: Determining z-score cutoff used in the ensemble LOINC code mapper**. The x-axis represents the z-score cutoff value used in the ensemble LOINC code mapper, and the y-axis represents corresponding precision.

**Supplementary Figure 2: Screenshot of RELMA.**
